# A way to thioacetate esters compatible with non-oxidative prebiotic conditions

**DOI:** 10.1038/s41598-020-71524-7

**Published:** 2020-09-02

**Authors:** Naoual Leqraa, Yvain Nicolet, Anne Milet, Yannick Vallée

**Affiliations:** 1 CNRS, DCM, Université Grenoble Alpes, Grenoble, France; 2 CEA, CNRS, IBS, Metalloproteins Unit, Université Grenoble Alpes, 38000 Grenoble, France

**Keywords:** Chemical biology, Chemistry

## Abstract

The centrality of pyruvate oxidative decarboxylation into acetyl-CoA in current biochemistry is a strong argument for proposing that a similar reaction have been necessary for the development of an effective protometabolism on the primitive Earth. However, such a decarboxylation requires the use of an oxidant and a catalyst, today enzymatic. Based on the mechanisms of the pyruvate dehydrogenase complex and pyruvate-ferredoxin oxidoreductase, we propose that the initial mechanism involved disulfides and occurred via radicals. A first disulfide is obtained by reacting glyoxylate with hydrogen sulfide. It is then possible to produce a wide variety of other disulfides by exchange reactions. When reacted with pyruvate under UV light they give thioesters. This process requires no oxidant and is therefore compatible with what is known of the redox conditions of the early Earth. Neither does it require any catalyst. It could be the first way to acetyl thioesters, a way that was later improved by the introduction of catalysts, first minerals, then enzymes.

## Introduction

The metabolism of living cells consists of a complex set of highly selective organic reactions, which are only possible thanks to the action of specific enzymes. Most of the reactions involved are however simple enough to postulate that during the setting up of the first stages at the origin of life, they already took place thanks to either simple catalysts, or even without any catalyst. Important proposals along these lines have been made recently. It is now possible, for example, to envisage the early development of the citric acid cycle (or reverse cycle)^1–6^, or the formation of highly activated intermediates such as phosphoenol pyruvate^7^, even though it has also been proposed that the primitive metabolism did not rely on phosphate groups^8^. Such a "phosphate-free world” was to resemble the thioester world initially described by Duve^9^. The process that we report here allows the oxidation of pyruvate into acetyl thioesters without requiring any catalyst. It exemplifies the possibility of a “catalyst-free protometabolism” and raises the idea that some parts of the nowadays metabolism were selected based on their intrinsic chemical properties rather than contingencies.

**Scheme 1:**
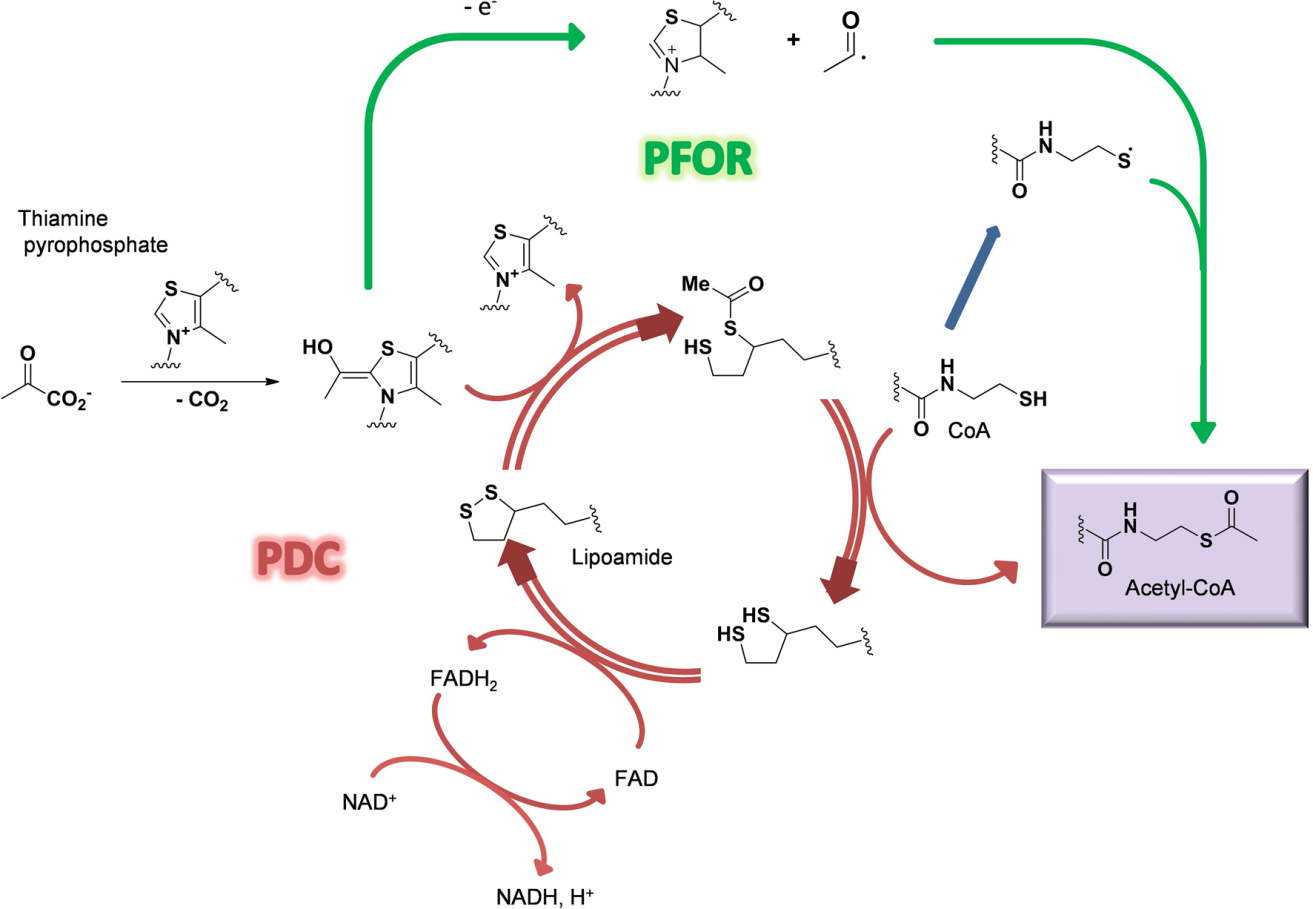
Simplified mechanism of PDC (red) and PFOR (green) decarboxylation pathways.

The emergence of life on Earth, despite being an ancient event, is still a hot topic as it relies on the possibility that life may have also occurred elsewhere in the Universe. Among the different hypotheses developed so far, two main schools of thought favour either information first as supported by the “*RNA-world*” hypothesis or the emergence of the metabolism prior to any information. Within the second current, several hypotheses have emerged leading to either a heterotrophic continuous metabolite supply, for instance from space, or the autotrophic development of metabolic loops that may have ultimately led to the known metabolism observed in all living organisms today. Lots of plausible, often fascinating, scenarios have been proposed, all facing similar difficulties^10–12^: What source of energy? What catalysts? For what selectivity? One concern regarding the heterotrophic hypothesis is that despite providing quite complex key molecules such as adenine^13^ or ribose^14^, it is difficult to understand how the transition would occur between such external supply and a subsequent need to develop synthetic paths even though such molecules already play a crucial role. One can also ask whether the present metabolism reflects legacies from how it has emerged and whether is it possible to turn the clock back. Have the main current metabolites been selected over many others due to intrinsic properties or because they are contingent of evolutionary events is another question. Pyruvate is most probably one of the key molecules at the heart of every living organism. Its detection in several meteorites^15^ and its possible synthesis under prebiotic conditions^2^ makes it a good candidate for a prebiotic actor. Pyruvate is usually associated to the formation of acetyl-coenzyme A (acetyl-CoA), a branching intermediate connecting the tricarboxylic acid cycle, the respiratory chain, the synthesis of amino acids and the production of lipids. The pyruvate dehydrogenase complex PDC is found in all kingdom of life. It contains three different enzymes and uses several cofactors to perform the oxidative decarboxylation of pyruvate, leading to acetyl-CoA and CO_2_ (Scheme 1)^16^. Acetyl-CoA can also be produced via the Wood-Ljungdahl pathway^17^, where two CO_2_ molecules are transformed into one carbon monoxide and a methyl. The carbon monoxide dehydrogenase CODH associated to the acetyl-CoA synthase ACS couple these two fragments and form an acetyl, which is subsequently bound to the thiolate moiety of CoA to produce acetyl-CoA. The pyruvate-ferredoxin oxidoreductase subsequently synthesizes pyruvate from acetyl-CoA and a third CO_2_ molecule. Interestingly, PFOR is often described as the ancestor of PDC and can also catalyse the reverse reaction producing acetyl-CoA from pyruvate^18^. The enzyme works under anaerobic conditions and uses radical-based chemistry. It contains a [Fe_4_S_4_] cluster and, like PDC, uses thiamine as a cofactor. Following the hypothesis that metabolisms has come first and was subsequently refined through the development and replacement of catalysts, we decided to address whether acetyl-CoA can be produced from pyruvate under prebiotic non-oxidant conditions. However, how could such an oxidation reaction occur in a non-oxidative world? Indeed, even though the idea that the primitive atmosphere of our planet was highly reductive has been abandoned by most authors (but not all of them^19^), it is still clear that it was not oxidative^20^. In the laboratory, such decarboxylation reactions are performed in the presence of strong oxidants, such as persulfates^21,22^.

Looking to nowadays biological mechanism in PDC (Scheme 1), it appears that the direct oxidant is lipoamide, outlining the importance of a disulfide bridge. Is it possible that disulfides have been used since the very beginning of the development of the protometabolism? If yes, is it possible to find a synthetic route for such disulfides following a plausible prebiotic non-oxidative process?

## Results and discussion

It has been reported that the reaction of glyoxylate with Na_2_S at 90 °C leads to a complex mixture of products containing some amount of dithiodiglycolic acid^23^. In this process two aldehydic carbon atoms are reduced, while two sulfur atoms, from sulfide anions, are oxidized with formation of a disulfide bridge and elemental sulfur. The presence of sulfur (as S_8_) was also evidenced when NaSH was reacted with dichloroacetic acid to yield mainly dithiodiglycolic acid and traces of trithiodiglycolic acid^24^. From a redox point of view, these reactions are neutral, and the reaction from glyoxylate can be considered as plausibly prebiotic^5,25,26^.

**Figure 1:**
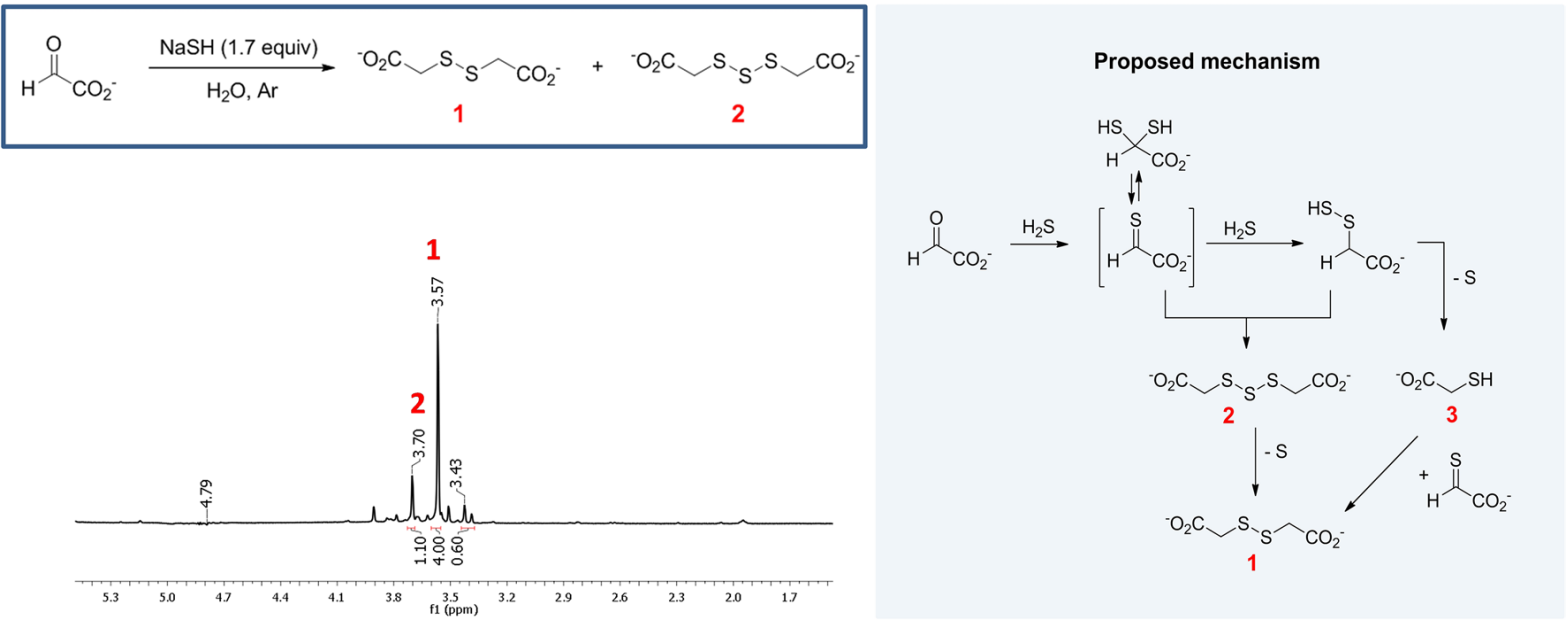
Synthesis of dithiodiglygolic acid **1**, ^1^H NMR spectrum of the reaction mixture, and proposed mechanism.

We have repeated this reaction under smoother conditions. Reactions were followed by ^1^H NMR. The spectrum of a representative reaction mixture is presented in Fig. 1. In this case, glyoxylic acid was reacted with 1.7 equiv. of NaSH in degassed water (pH *ca.* 6), under argon, at 70 °C. The major observed product is dithiodiglycolate **1**. Trithioglycolate **2** (yields **1**/**2**: 53/13) and some other by-products were also detected (Supporting Information, Scheme 1).

**Figure 2:**
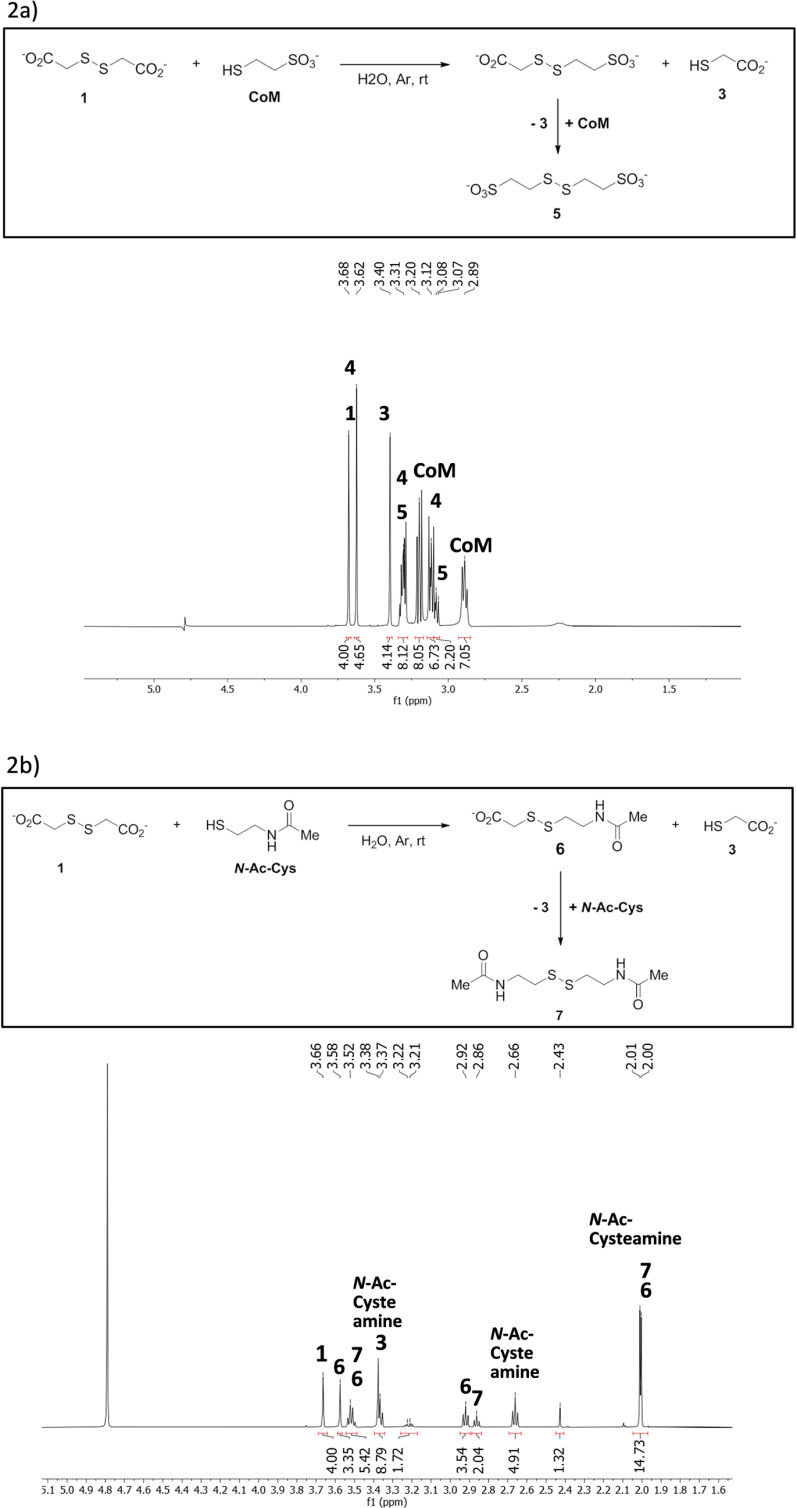
^1^H NMR spectra of exchange reactions. (**a**) Coenzyme M—dithiodiglycolic acid. (**b**) *N*-Acetylcysteamine—dithiodiglycolic acid.

It is thus possible that disulfide-containing compounds were produced this way on the early Earth, from various aldehydes^27^, but this not necessary. As soon as a disulfide is present, many others can be produced by the well-known thiol–disulfide exchange reaction^28–30^. These exchanges are classically promoted by bases. However, we decided to test them with simple mixtures of thiol and disulfide. Figure 2a shows the ^1^H NMR spectrum of a reaction mixture in which dithiodiglycolate **1** was mixed with coenzyme M (CoM) in degassed water (pH *ca.* 2) at room temperature. The spectrum was recorded after 7 days, a time sufficient for the ratios of products to reach equilibrium. It clearly shows thiol **3**, resulting from the reduction of **1**, and CoM disulfide **5**. The singlet at 3.62 ppm was attributed to the unsymmetrical disulfide **4**. The measured molar ratio of disulfides **1**, **4** and **5** is 37/43/20 (no other products were observed).

In addition to its present biochemical role^31^, CoM was chosen because of its higher solubility in water, but the reaction worked also with other thiols. Figure 2b presents a spectrum of the reaction with *N*-acetyl-cysteamine after 4 days in degassed water (pH *ca*. 2) at room temperature. The expected symmetric disulfide **7** was clearly observed, as was the non-symmetric *N*-acetyl-cysteamine—thioglycolate disulfide **6** with a ratio **1**/**6**/**7**: 42/37/21 (yields: **6** 33%, … **7** 19%).

Other tested thiols were mercaptoethanol and dithiothreitol. The exchange was observed in all cases (Supplementary Information spectra E and F). With dithiothreitol the unsymmetrical disulfide was not observed.

**Figure 3:**
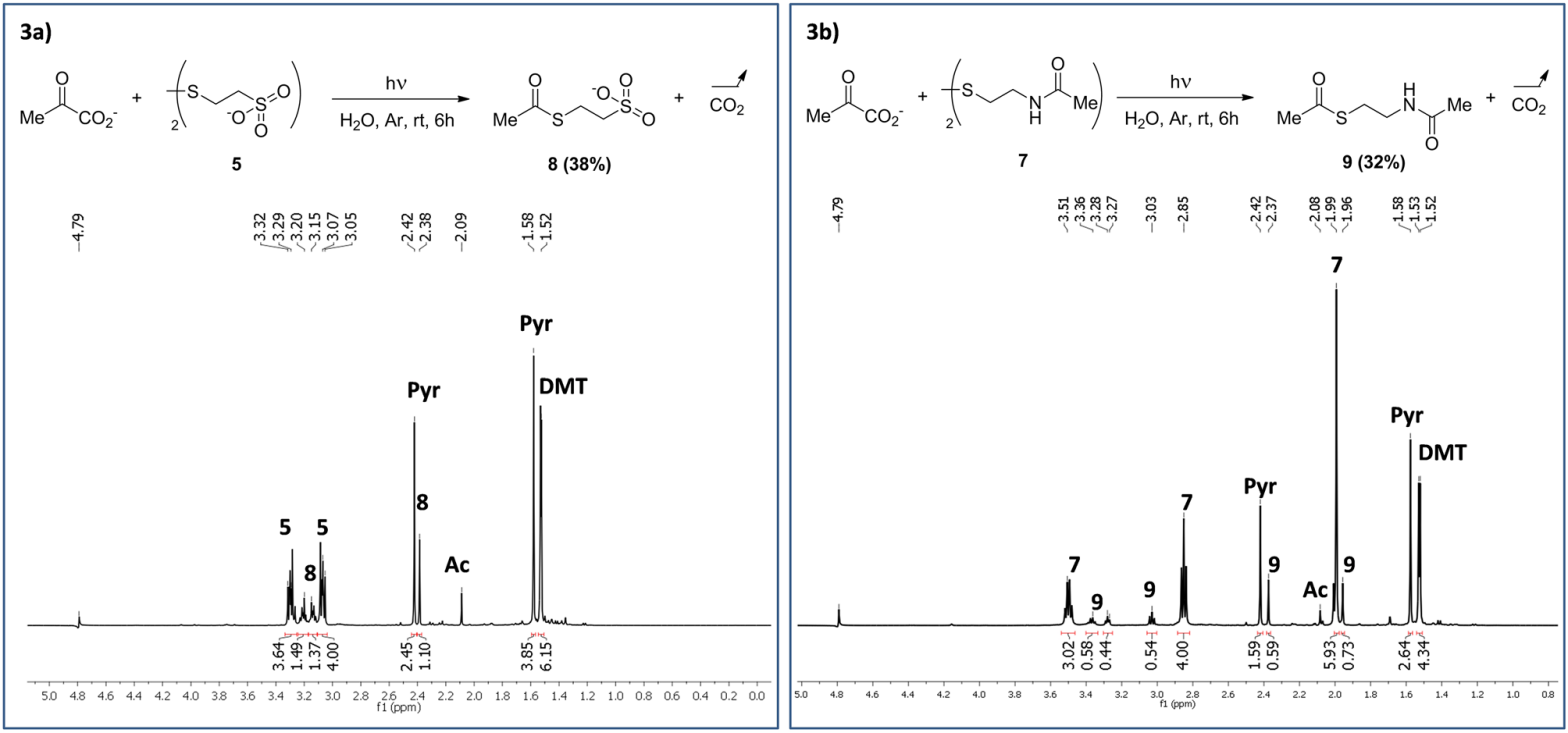
^1^H NMR spectra of the decarboxylation of pyruvate. 3a, with CoM disulfide **5**. 3b, with *N*-acetylcysteamine disulfide **7**.

For the pyruvate oxidative decarboxylation step, we choose to test CoM disulfide **5**, which has the advantage of being highly soluble in water, and *N*-acetylcysteamine disulfide **7**, reasonably soluble in water. The latter can be considered as a “simplified” CoA disulfide. In both cases, no reaction occurred upon heating, but when reaction mixtures were irradiated with UV light (pH of both reactions *ca.* 3), the expected thioesters **8** (yield 38%) and **9** (32%) were formed (Fig. 3).

The reaction with **7** was repeated at pH’s varying from 2 to 12, with various disulphide—pyruvate ratios (Supplementary Information table 1). Each time, thioester **9** was observed (range from 12 to 38% determined by ^1^H NMR, see Table 1 in Supporting Information). In few reactions we also identified another product, 2,3-dimethyltartrate (DMT), a classical reduction product of pyruvate (up to 31% of the starting pyruvate, which was always used in excess, was transformed into DMT), and, for the reaction starting with **7**, we assigned the signal at 3.26–3.29 ppm to a degradation product of *N*-acetylcysteamine disulfide, the hydrated form of acetamido-acetaldehyde^32^.

**Scheme 2:**
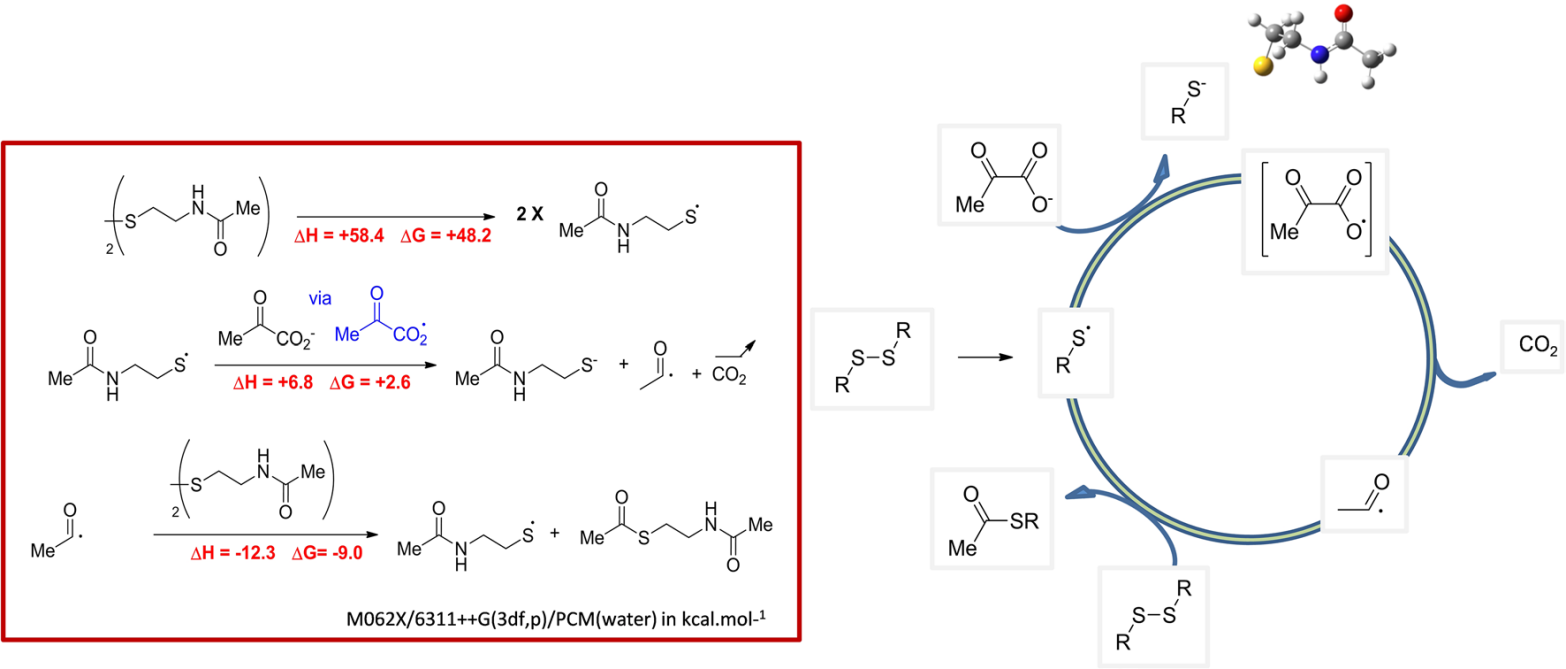
Proposed mechanism for the oxidative decarboxylation reaction of pyruvic acid under UV irradiation.

As it is induced by UV light, and as the formation of dimethyltartrate can be explained only by a pinacol coupling^33^, the mechanism of the decarboxylation reaction must involve a radical process. In the mechanism that we propose (Scheme 2), the initiation step is the cleavage of the S–S disulfide bond. Then, the obtained thiyl radical would abstract one electron from pyruvate, leading to a carboxyl radical. We subsequently performed theoretical calculations to assess the reliability of such mechanism. Several levels of theory were tested, notably to correctly describe the strength of the sulfur–sulfur bond, leading to the use of the M06-2X/6-311++g(3df,p) base in conjunction with an implicit solvent for water using the IEFPCM (integral equation formalism Polarizable Continuum Model) model. As already known on other examples^34^, the S–S bond dissociation requires *ca.* 60 kcal mol^−1^. Interestingly, the pyruvate carboxyl radical was predicted to be unstable, being easily cleaved to produce carbon dioxide and the acyl radical. The energetic cost of the reaction from thiyl radical + pyruvate to thiolate + CO_2_ + acyl radical was found to be low (6 kcal mol^−1^). Finally, the acyl radical would spontaneously react with the starting disulfide leading to the expected thioester and a new thiyl radical ready for another cycle. This step is exothermic (− 12 kcal mol^−1^).

## Conclusion

**Scheme 3:**
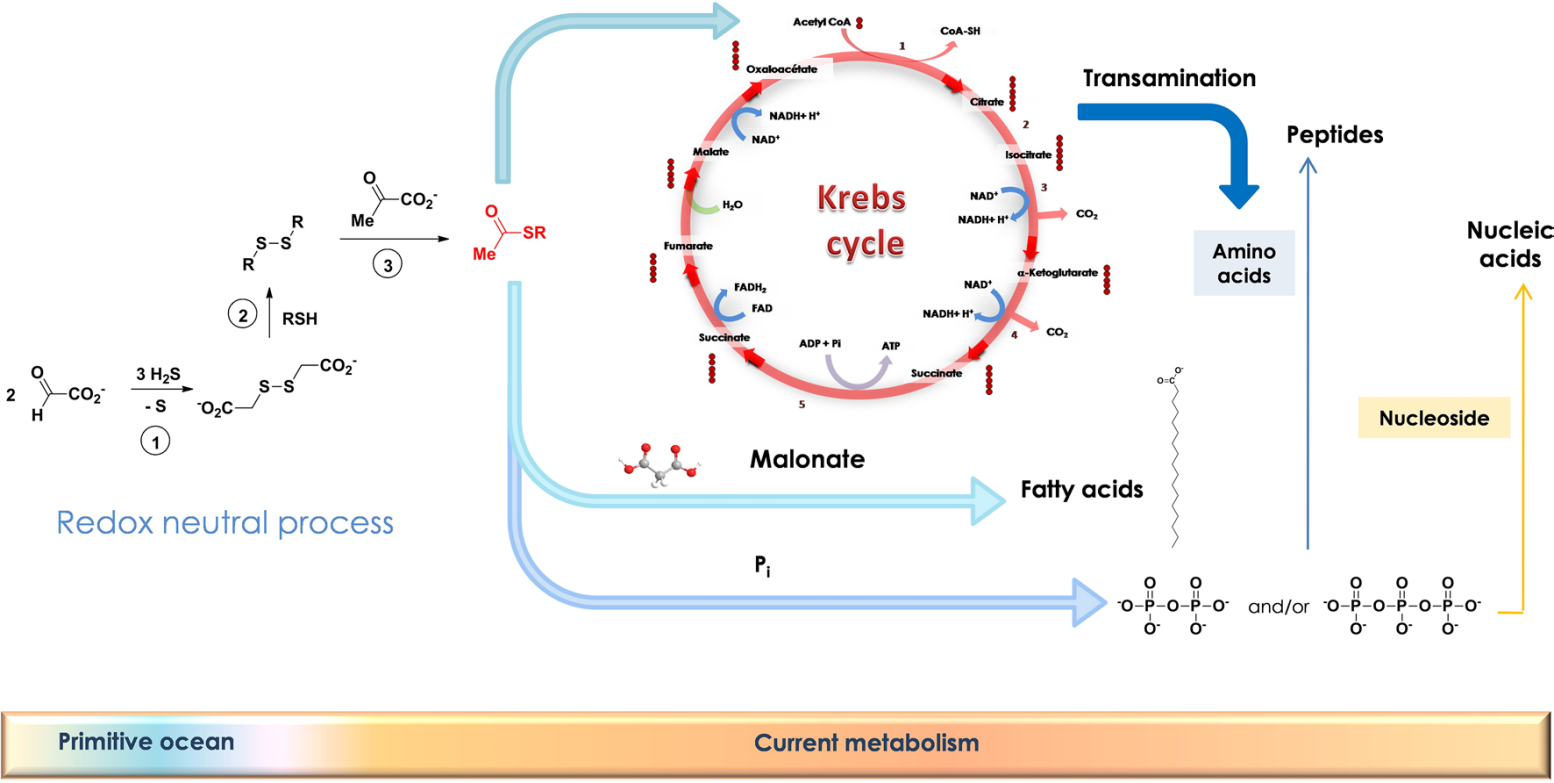
Global process leading to thioacetate esters and opening the way to various pathways of a protometabolism close to today’s one.

We have thus demonstrated that disulfides may have been synthesized in the non-oxidant primitive ocean (Scheme 3). Using dithiodiglycolate, obtained from glyoxylate, many other disulfide-containing compounds could be obtained and would, in turn, induce the oxidative decarboxylation of pyruvate. The resulting thioesters would be ready to enter the (proto-) citric acid cycle. It would also be used as energy source to obtain di- and triphostates^35^, hence polypeptides and nucleic acids. It is noteworthy that no catalyst was used for the three steps from glyoxylate to thioester, outlining some intrinsic properties of these compounds to perform such reactions observed in the current metabolism.

Over time this process may have improve with the introduction of catalysts, the first ones being probably simple minerals^36,37^, which were replaced by more elaborated metallic complexes, then proto-enzymes (probably proto-PFOR’s), at a time when primitive co-enzymes already existed. CoA itself was probably introduced as early as this proto-PFOR stage as was thiamine pyrophosphate, still acting under a radical mechanism. Then lipoamide was selected and introduced in a proto-PDC non-radical pathway. We are now considering coupling all these reactions over several diurnal cycles to test whether thioester accumulates, as well as its stability over time under UV-exposure^38^.

Our results pave the way to overcome the apparent need of polyphosphate to convey energy in the current metabolism and suggest thioesters to be good candidates for an ATP precursor in solution without requiring any surface chemistry. Thioesters are intermediates in the synthesis or polyketide and non-ribosomally-synthesized peptides^39,40^. Our findings suggest they could have also played this role on the early Earth, before being supplanted by the ribosome machinery.

## Method

All prebiotic (anaerobic) reactions were carried out under an inert atmosphere of argon, most often in NMR tubes. Irradiations of decarboxylation reactions in water were realized with a Hanau TG 150 high-pressure mercury arc lamp, generally in Pyrex NMR tubes (transparent from 320 nm). No significant difference was noticed when these reactions were run in quartz UV cells.

NMR spectra were recorded on Bruker AV 500 or AV 400, spectrometer at 500 MHz or 400 MHz for ^1^H NMR, at 125 or 100 MHz for ^13^C NMR.

Conversions were calculated from NMR spectrum, as were yields (using THF, 1,4-dioxane or acetic acid as quantitative standard).

Electrospray mass spectra were recorded on an ESI Ion Trap mass spectrometer BRUKER amaZon speed. High Resolution Mass Spectra were recorded on a LTQ Orbitrap XL Thermo Scientific.

### Synthesis of dithiodiglycolic acid

A NMR tube was charged with glyoxylic acid monohydrate (0.11 mmol), and sodium hydrosulfide (0.19 mmol). The NMR tube was evacuated under high vacuum and backfilled with argon. Degassed water (0.7 mL) was added, and the reaction mixture was heated for a night at 45 °C or 70 °C. 0.3 mL of D_2_O was added to realize the NMR experiment.

### Thiol–disulfide exchanges

A NMR tube was charged with dithioglycolic acid (0.044 mmol), and chosen thiol (0.088 mmol). The NMR tube was evacuated under high vacuum and backfilled with argon. Degassed water (0.7 mL) was added, and the reaction mixture was left at room temperature and followed by NMR.

### Decarboxylation reaction under UV light

A NMR tube was charged with disulfide (0.03 mmol), and pyruvic acid (0.09 mmol). The NMR tube was evacuated under high vacuum and backfilled with argon. Degassed water (0.7 mL) was added, and the reaction mixture was exposed to UV light for 6 h at room temperature.

## Supplementary information

Supplementary Information.

## Data Availability

The data sets and analysis of current study are available from the corresponding author upon reasonable request.
