# Supplementary material for: A way to thioacetate esters compatible with non-oxidative prebiotic conditions
Source: Sci Rep. 2020 Sep 2;10:14488. doi: 10.1038/s41598-020-71524-7 (PMC7467925; doi:10.1038/s41598-020-71524-7)
Supplement: Supplementary file 1 — Supplementary Information. [file 41598_2020_71524_MOESM1_ESM.docx]

**A way to thioacetate esters compatible with non-oxidative**

**prebiotic conditions**

Naoual Leqraa^1,2^, Yvain Nicolet^2^, Anne Milet^1^ & Yannick Vallée^1^

^1^ DCM, Univ. Grenoble Alpes, CNRS, Grenoble, France

^2^ IBS, Univ. Grenoble Alpes, CEA, CNRS, Grenoble, France

Correspondence and requests for materials should be addressed to Y.V.

(email: Yannick.vallee@univ-grenoble-alpes.fr)

**Contents**

**1 NMR spectra**

1. Dithiodiglycolic acid synthesis ………………………………………………………………………………………. 2
2. Trisulfide (compound **2**) ……………………………………………………………………………………………….. 3
3. Thiol – disulfide exchange of coenzyme M disulfide with dithiodiglycolate …………………… 4
4. Thiol – disulfide exchange of N-acetylcysteamine disulfide with dithiodiglycolate ………… 5
5. Thiol – disulfide exchange of N-acetylcysteamine disulfide with 2-mercaptoethanol ……..6
6. Thiol – disulfide exchange of N-acetylcysteamine disulfide with Dithiothreitol …………….. 7
7. Decarboxylation reaction using Coenzyme M disulfide …………………………………………………. 8
8. Decarboxylation reaction using Coenzyme *N*-Acetylcysteamine …………………………………. 10

**2 Tables**

Table 1: decarboxylation reaction of pyruvate at different pH ………………………………………….. 12

Table 2: Mass data…………………………………………………………………………………………………………......12

**3 Theoretical studies** ………………………………………………………………………………………………………… 13

**1 NMR** **spectra**

1. Dithiodiglycolic acid synthesis; pH *ca.* 6

Reaction at 45 °C


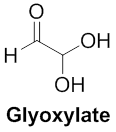

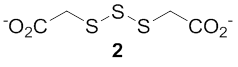

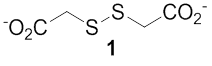


**Z**

**Glyoxylate**

**X**

**1**

**2**

**Y**

Reaction at 70 °C

**X**

**Y**

**2**

**1**

1. Trisulfide

Preparation of compound **2**: Harpp, D. N. & Smith, R. A. Reaction of trialkyles phosphites with organic trisulfides. Synthetic and mechanistic aspects. *J. Org. Chem*. **44**. 4140-4144

**2**

Tentative structure for compound **X**:

We suggest compound **Y** giving a singlet at 3.90 ppm is the tetrasulfide, and compounds **Z** (reaction at 45°C) giving signals between 5.22 and 5.52 ppm are thioacetal or dithioacetal compounds. **Z** compounds disappear at 70 °C while **X** and **Y** decrease.

1. Thiol – disulfide exchange of coenzyme M disulfide with dithiodiglycolate; pH *ca.* 2


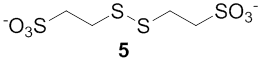

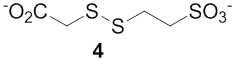


**1**

**4**

**3**

**5**

**CoM**

**4**

**5**

**CoM**

**4**

**3**

**4**

**14**

**CoM**

**4**

**5**

**1**

**5**

**4**

**3**

**CoM**

**1**

**3**

**4**

**CoM**

**CoM**

**3**

**5**

**1**

**4**

**4**

**5**

1. Thiol – disulfide exchange of N-acetylcysteamine disulfide with dithiodiglycolate; pH *ca.* 2


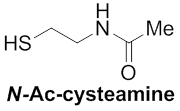

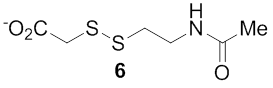

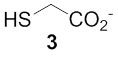

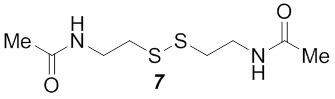


***N*-Ac-cysteamine**

***N*-Ac-cysteamine**

***N*-Ac-cysteamine**

**1**

**6**

**7**

**6**

**3**

**7**

**6**

**7**

**6**

**6**

**1**

**7**

***N*-Ac-cysteamine**

**3**

**7**

**6**

**6**

**7**

**1**

***N*-Ac-cysteamine**

***N*-Ac-cysteamine**

**3**

**7**

***N*-Ac-cysteamine**

1. Thiol – disulfide exchange of N-acetylcysteamine disulfide with 2-mercaptoethanol; pH *ca.* 2


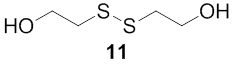

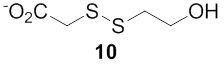

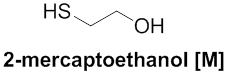


**11**

**10**

**10**

**[M]**

**[M]**

**11**

**10**

**3**

**1**

**3**

**10**

**1**

**1**

**3**

**[M]**

**[M]**

**11**

**10**

**10**

**10**

**11**

1. Thiol – disulfide exchange of N-acetylcysteamine disulfide with Dithiothreitol; pH *ca.* 2


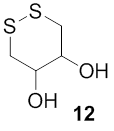


**1**

**1**

**3**

**3**

**12**

**12**

**12**

**12**

**12**

**3**

**1**

1. Decarboxylation reaction using Coenzyme M disulphide; pH *ca.* 3


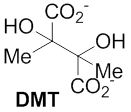

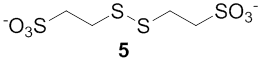

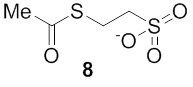

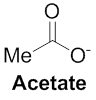


**Acetate**

**DMT**

**Pyr**

**Pyr**

**8**

**5**

**5**

**8**

**8**

**Pyr**

**8**

**Pyr**

**DMT**

**Pyr**

**Pyr**

**Pyr**

**DMT**

**5**

**8**

**5**

**8**

**Pyr**

**8**

**DMT**

2,3-Dimethyltartrate (DMT) : MARTIN, C., SERVAT, K., HUSER, H & KOKOH, K. B. Electrosynthesis of 2,3-dimethyltartaric acid from pyruvic acid in acid medium. *J. Appl. Electrochemistry*. **36**, 643-647 (2006).

Compound **8**: an authentic sample was prepared from 2-sulfanylethanesulfonate (Coenzyme M) and acetic anhydride.

Authentic ^1^H NMR

**5**

**5**

**5**

**8**

**8**

**8**

**8**

**8**

**8**

Reaction ^1^H NMR

1. Decarboxylation reaction using Coenzyme *N*-Acetylcysteamine; pH *ca.* 3


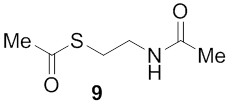


**DMT**

**7**

**Pyr**

**X’**

**X’**

**Acetate**

**Pyr**

**9**

**9**

**7**

**7**

**9**

**9**

**DMT**

**7**

**9**

**Pyr**

**Pyr**

**9**

**9**

**7**

**7**

**9**

**X’**

**DMT**

**X’**

**Pyr**

**9**

**7**

**DMT**

**Pyr**

**Pyr**

**Pyr**

**9**

Proposed structure for compound **X’**:

Compound **9**: An authentic sample was synthesized from N-Acetylcysteamine and acetic anhydride.

Authentic ^1^H NMR of compound **9**

**X’**

**X’**

**DMT**

**9**

**9**

**7**

**7**

**9**

**9**

**Pyr**

**9**

**9**

**Pyr**

**7**

**9**

**9**

Reaction ^1^H NMR

**2 Tables**

Table 1: decarboxylation reaction of pyruvate (Pyr) at different pH

| Entry | Pyr (equiv) | pH | **9** from **7** (%) | DMT from Pyr (%) |
| --- | --- | --- | --- | --- |
| **1** | 3 | 2 | 22 | 31 |
| **2** | 3 | 7 | 12 | 0 |
| **3** | 3 | 12 | 13 | 0 |
| **4** | 10 | 2 | 26 | 16 |
| **5** | 10 | 7 | 31 | 0 |
| **6** | 10 | 12 | 28 | 0 |
| **7** | 20 | 2 | 38 | 25 |
| **8** | 20 | 7 | 25 | 0 |
| **9** | 20 | 12 | 29 | 0 |

Table 2: Mass data

| **Compound** | **Calculated** | **Found** |  |
| --- | --- | --- | --- |
| **1** | 180.96347 | 180.96373 | C­_4_H_5_O_4_S_2_ [M-H] |
| **2** | 212.93554 | 212.93505 | C­_4_H_5_O_4_S_3_ [M-H] |
| **4** | 230.94611 | 230.94626 | C_4_H_7_O_5_S_3_ [M-H] |
| **5** | 280.92874 | 280.92883 | C_4_H_9_O_6_S_4_ [M-H] |
| **6** | 208.00966 | 208.01075 | C_6_H_10_O_3_NS_2_ [M-H] |
| **7** | 237.07260 | 237.07273 | C_8_H_17_O_2_N_2_S_2_ [M+H] |
| **8** | 182.97912 | 182.97951 | C_4_H_7_O_4_S_2_ [M-H] |
| **9** | 162.05833 | 162.05831 | C_6_H_12_NO_2_S [M+H] |

For carboxylic and sulfonic acids calculated masses are those of the protonated forms -H (negative ionisation).

**3 Theoretical calculations**

Following ref^1^, we tested different and recent functionals with or without dispersion correction^2^ and introduced the solvent effect through an implicit continuum using the IEFPCM (integral equation formalism Polarizable Continuum Model)^3^ or when otherwise stated its SMD variant^4^. These calculations were followed by a frequency calculation in the harmonic approximation to test the nature of the compounds and derived the thermodynamic data. The results obtained for MeSSMe are gathered in Table 1. As previously noted by Denk^1^, the B3LYP^5^ functional overestimates the S-S bond distance and logically underestimate the strength of sulfur-sulfur bond. Using dispersion corrections does not change the structural tendency but improves the bond dissociation enthalpy. On the other hand, the M06-2X^6^ functional gives better results and in conjunction a good quality basis set, the structural and energetical parameters are closer to the experimental results and the results obtained at the CBS and CBS-Q level with still a slight underestimation of the S-S bond enthalpy. These calculations were performed using Gaussian16B0.1^7^. These tests lead us to study the reactivity of N-acetylcysteamine disulfide using the M06-2X/6-311++g(3df,p) level of theory in conjunction with an implicit solvent using the IEFPCM model.

| MeSSMe | dS-S in Å | dS-C in Å | C-S-S in ° | XSSX in ° | ∆H(298.15K) |
| --- | --- | --- | --- | --- | --- |
| Exp. (MW=microwave)^8^ | 203.80 | 181.10(5) | 102.8 | 84.7 |  |
| ED^9^ | 202.9±0.3 | 181.6±0.3 | 103.2±0.2 | 85.3±3.7 |  |
| G3/CBS-Q^1^ |  |  |  |  | 63.07/64.78 |
| B3LYP/631+G** | 208.31 | 183.63 | 103.4 | 87.3 | 51.17 |
| B3LYP/6311G(2d,p) | 207.83 | 183.29 | 103.4 | 87.0 | 52.83 |
| B3LYP/6311++G(2d,p) | 207.81 | 183.30 | 103.6 | 87.5 | 52.95 |
| B3LYP/6311G(3df,p) | 205.34 | 182.32 | 103.9 | 87.0 | 55.91 |
| B3LYP/6311++G(3df,p) | 205.36 | 182.36 | 104.0 | 87.2 | 54.42 |
|  |  |  |  |  |  |
| M062X/6-31+G** | 206.07 | 181.98 | 101.9 | 84.9 | 57.85 |
| M062X/6311G(2d,p) | 205.67 | 181.74 | 101.7 | 84.2 | 61.23 |
| M062X/6311++G(2d,p) | 205.68 | 181.75 | 101.8 | 84.7 | 59.81 |
| M062X/6311G(3df,p) | 203.53 | 181.00 | 102.3 | 84.3 | 62.87 |
| M062X/6311++G(3df,p) | 203.56 | 180.99 | 102.3 | 84.5 | 61.79 |
| M062X/aug-ccpVTZ | 204.67 | 181.44 | 102.0 | 84.8 | 61.63 |
| M062X/6311++G(3df,p)/PCM (water) | 203.86 | 180.96 | 102.7 | 83.2 | 61.88 |
|  |  |  |  |  |  |
| B3LYP-D3/6311G(2d,p) | 207.79 | 183.46 | 102.8 | 84.7 | 56.55 |
| B3LYP-D3/6311G(3df,p) | 205.26 | 182.52 | 103.4 | 84.7 | 58.15 |
|  |  |  |  |  |  |
| B3LYP-D3BJ/6311G(2d,p) | 207.55 | 183.12 | 102.6 | 84.9 | 58.50 |
| B3LYP-D3BJ/6311++G(2d,p) | 207.55 | 183.09 | 102.8 | 85.4 | 57.10 |
| B3LYP-D3BJ /6311G(3df,p) | 205.10 | 182.12 | 103.2 | 85.0 | 60.11 |
| B3LYP-D3BJ /6311++G(3df,p) | 205.11 | 182.16 | 103.2 | 85.3 | 60.08 |

**Table 1:** Structural parameters and S-S bond dissociation for MeSSMe (distance in Å and angles in degrees, ∆H in kcal.mol^-1^). Experimental data from gas phase electron diffraction (ED), microwave (MW) studies. ∆H values are in kcal.mol^-1^.

For N-Acetylcystemaine disulfide, two conformers were found: one with a folded structure and the other one with a linear structure. The folded structure is stabilized by intramolecular hydrogen bonds but the lack of explicit water molecules in the solvent model complicates the comparison between the two conformers. So QM/MM^10^ dynamics was performed starting from the folded structure using CP2K software^11^. N-Acetylcystemaine disulfide was described at the BLYP level with a DZVP-MOLOPT-SR-GTH basis set in conjunction with GTH pseudopotentials. Dispersion was added by the empirical dispersion correction^2^. Water was described by a TIP3 model^12^ and was included through a cubic box of 15 Å around the molecule leading to the addition of 1750 water molecules. The GEEP scheme^13^ was used to calculate the QM/MM interactions. The QM cell has the size of the QM subsystems plus a distance of 8 Å on every direction. A cut-off of 400 Ry was retained to project the electronic structure. Periodic boundary conditions were applied to the MM and the QM system^14^. The time step for QM/MM simulations is 0.5 fs in the NVT ensemble with a CSVR thermostat with a 50 fs^-1^ constant.

This system was generated using amber18 software using gaff (generalized amber force field) parameters for N-Acetylcysteamine disulfide derived from the RESP^15^ approach using the electrostatic potential sampled in the MK scheme. Then it was minimized, followed by heating for 25 ps to reach an equilibrium temperature of 300 K, using a Langevin thermostat and a friction constant of 5 fs^−1^ in the NVT ensemble. This step was followed by 475 steps of equilibration in the NPT ensemble to ensure a correct density of 1. During this set-up the geometry of N-Acetylcystemaine disulfide was kept slightly fixed using a harmonic constraint with a global force constant of 200 kcal mol ^−1^ Å^−2^.

After 18000 steps the intra-molecular H-bond between the amide NH and the carbonyl CO (see Figure 1) are replaced by intermolecular H-bonds with water, which are excepted to be similar for the disulfide compound and its monomer. The promptness of this process confirms that the stability of N-Acetylcysteamine disulfide in a folded conformation is an artefact due to the absence of explicit water molecules. In the main body of the article, the values presented were therefore obtained from the data on the unfolded conformation.

|  | dS-S in Å | dS-C in Å | CSSC in ° | ∆H(298.15K) | ∆G(298.15K,*SMD*) |
| --- | --- | --- | --- | --- | --- |
| Linear | 203.5 | 182.5 | 87.8 | 58.40 | 48.15/*46.25* |
| Folded | 203.5 | 181.5 | 83.2 | 65.78 | 50.06/4*9.10* |

**Table 2:** Structural parameters and S-S bond dissociation for N-Acetylcysteamine disulfide (distance in Å and angles in degrees, ∆H and ∆G in kcal.mol^-1^, optimized structures at the M06-2X/6-311++g(3df,p)/PCM level of theory. In Italic, the values for ∆ﬂ obtained at the same level but using the SMD option in the PCM part of the calculation.

**
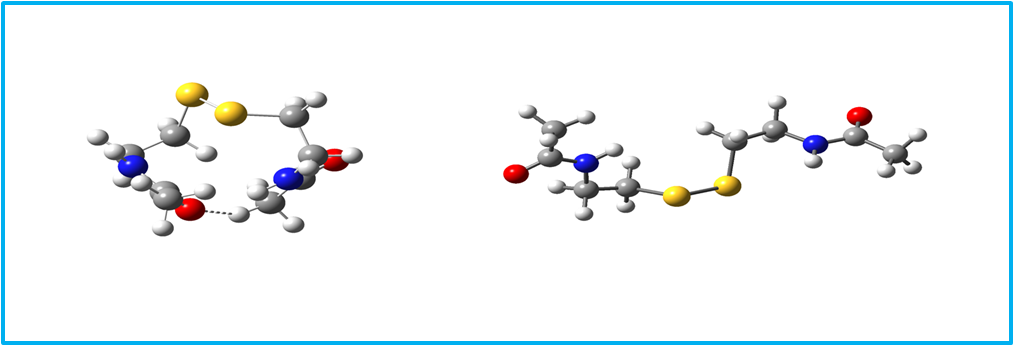
**

**Figure 1:** Linear and folded optimized structures of N-Acetylcysteamine disulfide at the M06-2X/6- 311++g(3df,p)/PCM level. H-bond of 1.965 Å in the folded conformation.


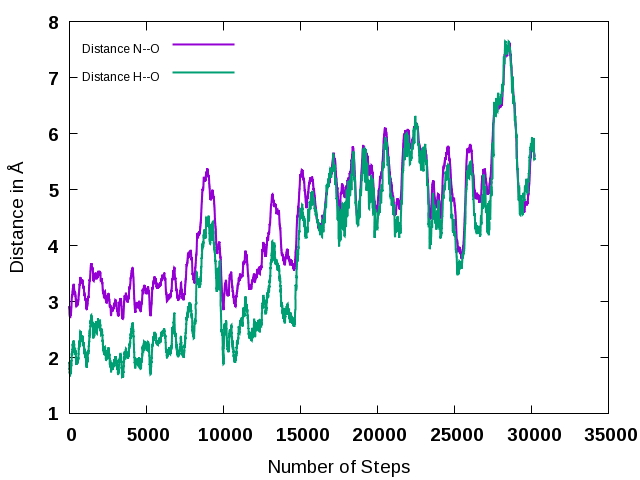
**Figure 2:** Evolution of the oxygen-nitrogen (initially involved in the intra-molecular H-bond) and of the hydrogen bond distance during the QM/MM dynamics

**References** (theoretical calculations)

1. Denk, K. M. *Eur. J. Inorg. Chem.* 1358–1368 (2009).
2. Grimme, S., Antony, J., Ehrlich, S. & Krieg, H. *J. Chem. Phys.* **132**, 154104 (2010). Grimme, S., Ehrlich, S. & Goerigk, L. *J. Comput. Chem.* **32**, 1456-1465 (2011).
3. Tomasi, J., Mennucci, B. & Cammi, R. *Chem. Rev.* **105** 2999-3093 (2005).
4. A. V. Marenich, C. J. Cramer & D. G. Truhlar. *J. Phys. Chem. B*, **113** 6378-96 (2009). doi: [10.1021/jp810292n](http://dx.doi.org/10.1021/jp810292n)
5. Becke, A.D., *J. Chem. Phys.* 98 (1993) 5648-5652. C. Lee, W. Yang, R.G. Parr, *Phys. Rev. B* ***37***, 785-789 (1988). S.H. Vosko, L. Wilk & M. Nusair, *Can. J. Phys.* **58**, 1200-1211 (1980). P.J. Stephens, F.J. Devlin, C.F. Chabalowski & M.J. Frisch, *J. Phys. Chem.* **98**, 11623-11627 (1994).
6. Zhao, Y. & Truhlar, D. G. *Theor. Chem. Acc.*, **120**, 215-41 (2008). doi: [10.1007/s00214-007-0310-x](http://dx.doi.org/10.1007/s00214-007-0310-x)
7. Gaussian 16, Revision B.01, Frisch, M. J., Trucks, G. W., Schlegel, H. B., Scuseria, G. E., Robb, M. A., Cheeseman, J. R., Scalmani, G., Barone, V., Petersson, G. A., Nakatsuji, H., Li, X., Caricato, M., Marenich, A. V., Bloino, J., Janesko, B. G., Gomperts, R., Mennucci, B., Hratchian, H. P., Ortiz, J. V., Izmaylov, A. F., Sonnenberg, J. L., Williams-Young, D., Ding, F., Lipparini, F., Egidi, F., Goings, J., Peng, B., Petrone, A., Henderson, T., Ranasinghe, D., Zakrzewski, V. G., Gao, J., Rega, N., Zheng, G., Liang, W., Hada, M., Ehara, M., Toyota, K., Fukuda, R., Hasegawa, J., Ishida, M., Nakajima, T., Honda, Y., Kitao, O., Nakai, H., Vreven, T., Throssell, K., Montgomery Jr., J. A., Peralta, J. E., Ogliaro F., Bearpark, M. J., Heyd, J. J., Brothers, E. N., Kudin, K. N., Staroverov, V. N., Keith T. A., Kobayashi, R., Normand, J., Raghavachari, K., Rendell, A. P., Burant, J. C., Iyengar, S. S., Tomasi, J., Cossi, M., Millam, J. M., Klene, M., Adamo, C., Cammi, R., Ochterski, J. W., Martin, R. L., Morokuma, K., Farkas, O., Foresman, J. B. & Fox, D. J. Gaussian, Inc., Wallingford CT, 2016.
8. Sutter, D., Dreizler, H. & Rudolph, H. D., *Z. Naturforsch. Teil A* **20**, 1676 (1965).
9. Beagley, B. & McAloon, K. T. *Trans. Faraday Soc.* **67**, 3216 (1971).
10. Warshel, A. & Levitt, M. *J. Mol. Biol.* **103**, 227-249 (1976).
11. VandeVondele, J., Krack, M., Mohamed, F., Parrinello, M., Chassaing, T., Hutter, J. *Comput. Phys. Commun.* **167**, 103-128 (2005). Hutter, J., Iannuzzi, M., Schiffmann, F., VandeVondele, J. *Wiley Interdiscip. Rev. Comput. Mol. Sci.* **4**, 15 (2014).
12. Jorgensen, W. L.; Chandrasekhar, J.; Madura, J. D.; Impey, R. W.; Klein, M. L. *J. Chem. Phys.* **79**, 926-935 (1983).
13. Laino, T., Mohamed, F., Laio, A. & Parrinello, M. *J. Chem. Theory Comput.* **1**, 1176-1184 (2005). Laino, T., Mohamed, F., Laio, A. & Parrinello, M. *J. Chem. Theory Comput.* **2**, 1370-1378 (2006).
14. Blöchl, P. E. *J. Chem. Phys.* **103**, 7422-7428 (1995).
15. Bayly, C. I., Cieplak, P., Cornell, W. & Kollman, P. A. *J. Phys. Chem.* **97**, 10269-10280 (1993).

**Optimized Cartesian Coordinates at the M06-2X/6-311++g(3df,p)/PCM-water level.**

Pyruvate

9

C 0.86878 -0.11941 0.00042

O 1.07855 -1.34692 0.00555

O 1.68564 0.81337 -0.00597

C -0.64973 0.28771 0.00048

O -0.97959 1.44800 0.00523

C -1.65542 -0.82921 -0.00446

H -1.49473 -1.46127 -0.87962

H -1.49849 -1.46549 0.86828

H -2.66527 -0.42340 -0.00577

Pyruvate radical (CO2+ acyl radial)

9

C -1.74944 0.03708 -0.00003

O -1.54619 1.17345 -0.00034

O -1.97359 -1.09463 0.00030

C 1.27191 -0.29444 0.00001

O 1.89476 -1.28949 -0.00028

C 1.73460 1.13238 0.00027

H 1.31651 1.62426 -0.87848

H 1.31663 1.62393 0.87926

H 2.82460 1.18704 0.00019

N-acetylcysteamine disulfide linear conformation

30

S 1.00245 -1.19869 0.02850

S -0.89804 -1.08418 0.74618

C 1.32934 0.52172 -0.48614

H 1.11403 1.17939 0.35681

H 0.67087 0.77446 -1.31592

C -1.90342 -1.29954 -0.76230

H -1.77205 -2.31760 -1.12428

H -1.55280 -0.59928 -1.52141

C 2.77519 0.65476 -0.92788

H 2.93483 1.66463 -1.31392

H 2.99503 -0.03989 -1.74400

C -3.36511 -1.05039 -0.43776

H -3.96826 -1.28713 -1.31695

H -3.69886 -1.71166 0.36839

N -3.58682 0.33569 -0.07154

C -4.83002 0.82591 0.09816

H -2.78952 0.92404 0.11936

O -5.82302 0.12935 -0.06762

N 3.67956 0.41223 0.18036

C 5.01331 0.53181 0.03573

H 3.29937 0.12392 1.06924

O 5.51577 0.85652 -1.03236

C -4.93383 2.27606 0.49749

H -5.46044 2.33526 1.45042

H -3.96570 2.76539 0.58926

H -5.53335 2.79812 -0.24860

C 5.85145 0.24749 1.25630

H 5.25924 -0.02805 2.12727

H 6.54389 -0.56076 1.01967

H 6.44044 1.13572 1.48624

N-acetylcysteamine disulfide folded conformation

30

S -0.78340 1.82774 -1.27779

S -0.47150 1.76793 0.73958

C -0.74050 0.09393 -1.81422

H 0.07504 -0.40188 -1.29252

H -0.49175 0.13526 -2.87722

C 1.34225 1.81369 0.94910

H 1.57403 2.67319 1.57968

H 1.78831 1.96810 -0.03310

C -2.05164 -0.66474 -1.63571

H -1.89986 -1.69436 -1.96963

H -2.82927 -0.21851 -2.25441

C 1.87372 0.53532 1.59085

H 2.93679 0.66789 1.80092

H 1.36314 0.34817 2.53658

N 1.71108 -0.62885 0.74890

C 2.56687 -0.89909 -0.25596

H 0.85585 -1.17162 0.81966

O 3.56015 -0.21595 -0.47769

N -2.53826 -0.67891 -0.26912

C -1.88705 -1.31994 0.71369

H -3.30851 -0.07669 -0.02577

O -0.89185 -2.00730 0.48787

C 2.23631 -2.11362 -1.08950

H 2.26376 -1.83222 -2.14259

H 3.00830 -2.86711 -0.92486

H 1.26127 -2.53460 -0.84533

C -2.42156 -1.15101 2.10967

H -2.58313 -2.13645 2.54637

H -1.65685 -0.64053 2.69820

H -3.34541 -0.57645 2.14791

N-acetylcysteamine radical

15

S -2.33346 -0.78049 -0.22345

C -1.45260 0.77556 -0.29935

H -2.19225 1.58058 -0.31578

H -0.94313 0.79989 -1.26833

C -0.44324 0.98752 0.82428

H 0.00882 1.97475 0.70952

H -0.94204 0.94787 1.79198

N 0.61085 -0.00457 0.81710

C 1.62249 0.04522 -0.07401

H 0.53310 -0.80275 1.42642

O 1.72208 0.95479 -0.88743

C 2.63002 -1.07449 -0.00894

H 3.61709 -0.64207 0.15669

H 2.41602 -1.79740 0.77636

H 2.64515 -1.58225 -0.97374

N-acetylcysteamine anion

15

S -2.54331 -0.70903 0.18164

C -1.74202 0.80246 -0.46671

H -2.37296 1.67326 -0.27794

H -1.62051 0.72055 -1.55065

C -0.37713 1.07784 0.15108

H 0.08985 1.94946 -0.31361

H -0.48792 1.28722 1.22136

N 0.49633 -0.06236 -0.04543

C 1.83288 0.00299 0.02432

H 0.02745 -0.95872 -0.09712

O 2.43856 1.05522 0.21386

C 2.57467 -1.30026 -0.15644

H 3.17636 -1.48415 0.73430

H 1.91258 -2.14783 -0.32637

H 3.25501 -1.19875 -1.00262

Acyl radical

6

C -1.16505 0.09778 -0.00002

H -1.17429 1.18936 -0.00076

H -1.67477 -0.29661 -0.87925

H -1.67412 -0.29531 0.88019

C 0.24279 -0.42355 -0.00002

O 1.25710 0.16965 0.00000

N-acetyl-S-acetylcysteamine

21

S -1.16387 -0.69809 -0.69324

C -0.20563 0.84049 -0.69915

H -0.86157 1.68554 -0.90156

H 0.47464 0.74119 -1.54590

C 0.60640 1.05956 0.57416

H 1.12068 2.01944 0.49395

H -0.03955 1.09208 1.45122

N 1.59432 0.02296 0.78663

C 2.74421 -0.01371 0.08047

H 1.38645 -0.72618 1.42688

O 3.01860 0.84330 -0.74895

C 3.67311 -1.16517 0.36989

H 4.65221 -0.76269 0.62970

H 3.32040 -1.80874 1.17391

H 3.78484 -1.75496 -0.54065

C -2.70643 -0.30856 0.10256

O -3.47439 -1.22001 0.27877

C -3.01724 1.11234 0.48144

H -2.23573 1.53834 1.11050

H -3.09416 1.72437 -0.41948

H -3.96684 1.12433 1.01205
